# The assessment of preschool children with ESSENCE symptoms: concordance between parents, preschool teachers and child psychologists

**DOI:** 10.1186/s12887-024-04693-3

**Published:** 2024-03-16

**Authors:** B. M. Gustafsson, M. Sund Levander

**Affiliations:** 1 Linköping University, Department of Child and Adolescent Psychiatry, Department of Biomedical and Clinical Sciences, Linköping, Sweden; 2 Psychiatric Clinic, Högland Hospital Eksjö, Department of Psychiatry and Rehabilitation, Region Jönköping County, Jönköping, Sweden

**Keywords:** Behaviour, Child health care, Everyday function, ESSENCE, Parents’, Preschool teachers’

## Abstract

**Background:**

It is important to detect children with Early Symptomatic Syndromes Eliciting Neurodevelopmental Clinical Examinations (ESSENCE) in order to implement early intervention and support for the child and family. Standardized instruments for assessment in different contexts of behaviour problems, engagement and psychosocial health obtain an objective picture of the preschool child's mental health.

**Aim:**

To explore and compare parents', preschool teachers' and child health care psychologists' assessment of behaviour, everyday function, engagement, social interaction and psychosocial health in children with ESSENCE symptoms.

**Method:**

Parents of 152 children (114 boys and 38 girls, 4.5 ± 1 years) with ESSENCE symptoms, 155 preschool teachers and 8 child psychologists participated. Parents and preschool teachers assessed externalizing and internalizing behavioural problems using the Strengths and Difficulties Questionnaire (SDQ), including the SDQ supplement for assessing the impact of behavioral problems on daily function. Preschool teachers also assessed engagement and social interaction using the Children's Engagement Questionnaire (CEQ), and the child psychologists assessed psychosocial health with the Child Psychosocial Health Assessment (LillaLAPS) and template in conversations with parents of children with neurodevelopmental problems.

**Results:**

Parents', preschool teachers' and child psychologists' assessment of the child's ESSENCE symptoms overall agreed. Both parents and preschool teachers see a strength in the child's social abilities. Differences in mean values show that parents assess more conduct, emotional symptoms and problems in daily life and more social skills, compared to the preschool teachers rating more peer problems.

**Conclusion:**

It is important to consider different contexts to identify the child's need for support in everyday life. Expanded use of validated screening instruments in clinical practice would promote detection of children not already identified as exhibiting neurodevelopmental problems.

## Introduction

It is important to detect children with neurodevelopmental problems in order to be able to implement early intervention and support for the child and family. With preschool children, it is difficult to get a clear picture of whether the child has neurodevelopmental problems because children develop differently in relation to biological age. Early Symptomatic Syndromes Eliciting Neurodevelopmental Clinical Examinations (ESSENCE) is a relative new approach for better understand neurodevelopmental symptoms. In addition to clinical anamnesis, standardized instruments for assessment of behaviour problems, engagement and psychosocial health are needed. It is also important to gather information from different contexts to gain an objective picture of the preschool child's mental health. This paper focus on parents', preschool teachers’ and child psychologists' assessment of children with ESSENCE symptoms, referred to a CHC psychologist.

## Background

A prerequisite for children with neurodevelopmental problems to have good mental health later in life is that the child receives the support of adults for learning to regulate and control emotions, attention, behaviour, cognition and independence [1, 2]. Early detection by screening is in line with Skovgaard et al. [3], who found that predictors of neuro-developmental illness and parent–child relationship disturbances could be identified in the first 10 months of life in children. There is also an economic benefit to early detection and thus the ability to prevent mental health problems in young children [4, 5].

The prevalence of mental illness in preschool children is reported to vary between 5% [6] and 17% [7]. However, it is often unclear whether an early illness is best interpreted as an expression of problematic interpersonal relationships or as an early symptom of individual mental illness. Von Kietzing et al. [7] emphasize the importance of acting cautiously when assigning psychopathological significance to symptoms arising in early childhood, but also managing to recognize mental illness early on from the way they are embedded in the child's interactive relationships with parents or significant others. Children with neurodevelopmental problems and externalizing/internalizing behavioural problems are at greater risk of mental illness later in life [8–10].

Engagement means the extent to which the child in preschool is actively involved in daily activities such as playing and learning activities by themselves or in social interaction with adults or other children [11]. Engagement can be described multidimensional as the child's behaviour, emotions and cognitive function [12, 13]. Among preschool children, engagement is a strong predictor for learning, sociability, mental health [14, 15], self-regulation and academic success [16].

Children have important development-related tasks to solve in various areas, such as motor skills, language and communication affect regulation and self-image. For this reason, it is important to have knowledge of what is "typical of development" for different ages in order to be able to detect development-related delays/deviations or behavioural problems at an early stage and offer adequate support [17].

The younger the children, the more difficult it is to distinguish between behaviours that are "typical" for the age and those that are deviant. For this reason, it is of great importance to have different sources of information and contexts where the behaviour is manifested to obtain an objective picture of the child's function [18]. The younger the age, the more the child's behaviour depends on relationships with guardians and family members. Judgment of whether a behaviour is due to a mental illness or just an expression of normality must be based on the child's current stage of development and personal characteristics [7].

Behavioural problems, including difficulties with self-regulation and temperament, can manifest themselves in a lack of language and communication skills [19], but can also be caused by other development-related difficulties [20]. Difficulties with emotion regulation tend to lead to internalization such as shyness and reluctance and/or externalization in the form of aggression, outbursts and antisocial behaviour [21]. Raspa et al. [15] also identified the child's involvement and social interaction in everyday activities as a predictor of mental illness.

Showing neurodevelopmental problems in early childhood is often associated with the presence of a Neurodevelopmental Disorders (NDD) and lifelong disability requiring support [22, 23]. The clinical presentation of NDD can show a spectra of neurodevelopmental problems, especially in early years, with symptoms in a variety of fields [22]. Cognitive skills involve thinking abstractly, solving problems, storing memories and experiences. Memory develops and affects the learning of new knowledge and communication in young children, while a good social function also includes well-developed language and communicative skills [24]. The emotional development of children includes understanding their own and others' feelings of joy, sadness, fear, anger, pride, shame, guilt and envy, as well as empathy and regulation of expressions of bodily reactions [25, 26]. Gillberg [23] emphasized that behavioural problems found in early-onset neuropsychology and developmental neurology may be overlapping and concurrent, in contrast to separate and of an ‘either or’ nature. Therefore, he coined the term Early Symptomatic Syndromes Eliciting Neurodevelopmental Clinical Examinations (ESSENCE) [23]. The concept is based on the fact that the symptoms of different diagnoses within the neuropsychiatric spectrum can be the same at the beginning of the child's life, which is why it is not always easy to make a diagnosis based on specific criteria. Rather than establishing a specific neuropsychiatric diagnosis, ESSENCE highlights comorbidity and includes clinical symptoms that the preschool child shows related to general development, behavior, attention, activity, social interaction, communication and language, motor coordination, mood and/or sleep. Children with one or more symptoms will be referred to interprofessional assessment by e.g. psychologist, specialist nurse, social worker, education specialist and paediatrician [23, 27, 28]. According to Gillberg [23], approximately 13% of Swedish preschool boys and 7% of girls present ESSENCE symptoms. In addition, boys are reported to have significantly more externalizing symptoms than girls and also considered to have significantly lower prosocial scale than girls [29, 30].

Ogundele [31] points out that neurodevelopmental problems can be understood in an balance between children's biological assets and contexts. With a holistic approach, i.e. identifying health factors in the child's social interaction, deviant behaviour can be detected earlier by screening [29], which increases the possibility of offering adequate support at an early stage [23, 32]. Early detection of ESSENCE symptoms promotes more effective intervention and reduces the risk of later mental illness and human suffering [33].

There are national guidelines for the use of validated psychometric instruments in the CHC psychologist assessment of children with behavioural problems, including ESSENCE symptoms. Various psychometric instruments are used in both primary childcare and specialist care regarding children's development/behaviours. However, there are differences regarding the choice and application of instruments in clinical practice. There is also a lack of studies of sufficiently good quality and sufficient data [34].

Efforts made to strengthen overall mental health in preschool children appear to lead to positive effects later in life [23]. However, during recent decades the number of children reported with mental health problems has increased in Sweden [35–37]. About 90% of Swedish children between the ages of 1 and 6 years attend preschool, which is thus a suitable context for detection to early identify mental health problems. According to the Swedish National Agency for Education [38], the preschool teacher is responsible for learning and the continuous development of the child, but it is the preschool activities themselves and not the individual child that are intended here. Praxis in Swedish preschools is not to screen for abnormal physical and cognitive abilities in individual children. Within the field of health care, the Child Health Care System (CHC) has a well-established program for prevention and early detection of physical and mental health problems among preschool children. The program, including 9 visits during the child's first year of life and then at 1.5, 2.5, 3, 4 and 5 years of age, reaches 95% of all preschool children in Sweden [39]. The Child Health Nurse (CHN) is the coherent link in the contacts [38]. If worried about a child's health also the preschool teacher may, with consent from parents or together with parents, contact the CHN in order to work together in matters concerning the child [40].

To prevent mental health problems later in life, one way is to detect ESSENCE symptoms by screening, and initiate support for both the child and their parents, it is crucial to gather information from the child's different contexts [23, 41]. In order to improve processes and identify and support preschool children’s overall needs in all systems, the Mental Health, Learning, Development, and Collaboration for Young Children (PLUSS) model was launched in 2019 in Jönköping County, Sweden and retrospectively registered in the Clinical Trials 2021, PLUSS identifier, NCT04815889 [42, 43]. The present study is a part of this project. In this project, the question of concordance in the assessment when standardized instruments are used by parents, preschool and clinical assessment by a psychologist has been brought up to date.

### Purpose

To explore and compare parents', preschool teachers', and the child health care psychologists' assessment of behaviour, everyday function, engagement, social interaction and psychosocial health in children with ESSENCE symptoms.

## Method

The study has an explorative, comparative design. Data was gathered between May 2019 and September 2021.

### Sample

We obtained the participants by recruiting a convenience sample of children in Jönköping County, Sweden [44, 45]. The inclusion criteria were children referred to a CHC psychologist after adults in vicinity of the child observed and raised concerns. The child was included after informed consent from the parents. The final sample consisted of 152 parents of 152 children (114 boys and 38 girls, 4.5 ± 1 years old, range 1.5 to 6.0 years), 8 child health care psychologists (median worked time in the CHC, 7 years, range 1–17) and 155 preschool staff of whom 89% had a preschool teaching degree. The remaining 11% were childcare workers and leisure educators. The median time the staff had worked in preschool was 20 years and 62% had known the child in question for more than 12 months.

Forty-eight percent of included parents had higher education and 8% primary school, 93% were employed or studied and 70% (101/152) had Swedish as their mother tongue. All included parents mastered the Swedish language without the need for help of an interpreter.

### Measurements

The included psychometric instruments aimed to assess externalizing and internalizing behavioural problems (SDQ), and engagement and social interaction (CEQ), alongside the Child Psychosocial Health Assessment (LillaLAPS) and clinical anamnestic psychologist's template in conversations with parents of children with neurodevelopmental problems.

### The Strengths and Difficulties Questionnaire (SDQ)

The SDQ is a brief behavioural screening questionnaire that can be used by parents and preschool staff. The SDQ consists of five subscales assessing emotional symptoms, conduct problems, hyperactivity and inattention, peer problems, and prosocial behaviour. The answers are given in 3 alternatives: 0 = not true, 1 = partly true, 2 = completely true [46–48]. Goodman found a specificity of 94.6% and a sensitivity of 63.3% in a British population of children aged 5 to 15 years [46, 48] while Gustafsson et al. [49] reported a high sensitivity, 97.4%, but a low specificity of 13.8% in preschool children. Borg et al. [50] reported good internal consistency, inter-rater and cross-informant agreements and test–retest of the method. However, they point out that the gender and age of the child, the number of informants and cultural differences in reporting styles affected the results and thus confirmed the need to re-evaluate the SDQ in the culture and population in question. Nevertheless, Gustafsson et al. [51] found the SDQ to be good enough to identify children with ESSENCE symptoms when the symptoms were assessed by preschool staff. The cut-off for behavioural problems in the SDQ differs between screening by parents and by preschool teachers, as parents usually score higher than preschool teachers [47]. Croft et al. [52] also reported SDQ subscales to have high sensitivity in differentiating children with ESSENCE symptoms from the normal population of children in Sweden.

### SDQ impact supplement

There is also an impact supplement consisting of questions about the child's behavioral problems impact on daily functions [47, 53]. The impairment supplement starts with the question "Overall, do you think that this child has difficulties in one or more of the following areas: emotions, concentration, behavior or being able to get on with other people?" If the preschool teacher or parents answered "Yes" to this question, they were asked to answer the question about these difficulties: "How long have these difficulties been present?", "Do the difficulties upset or distress your child?" and "Do the difficulties interfere with the child's everyday life in the following areas. The range is 0–8 for preschool and 0–10 for parents, ratings of "Not at all" and "Only a little" were scored as 0, "Quite a lot" as 1 and "A great deal" as 2 [47].

### The Children's Engagement Questionnaire (CEQ)

Engagement and social interaction were assessed in the preschool context using the CEQ, which is designed to be used by preschool teachers. It was developed by McWilliams [54] and later adjusted to a Swedish context by Almqvist [55]. The original CEQ consists of 32 items with four underlying factors: competence, persistence, undifferentiated behavior, and attention [56]. The Swedish version of the CEQ consists of 29 items, since three of the items were judged not to be relevant in the Swedish preschool context [57]. The answers are given in 4 alternatives: 1 = almost never happens, 2 = sometimes happens, 3 = happens quite often, 4 = happens very often. The CEQ is widely used in educational research in Sweden and shows good measurement properties [49, 55, 57]. In a normal population of Swedish preschool children, the CEQ mean value is reported to be 3.2 + 0.61 [29, 30]

### The Child Psychosocial Health Assessment (LillaLAPS)

LillaLAPS is a recently developed Finnish instrument to assess the psychometric properties by health care professionals in the context of mental health care for children [58]. The instrument consists of 18 items about development related to age, somatic diseases, everyday function, physical/mental/social ability, internalizing/externalizing behavior, parents' ability to regulate the child's behavior and emotions, substance abuse, domestic violence and parent's anxiety. The answers are given in 3 alternatives: 0 = no/not present, 1 = minor, 2 = moderate or severe. Total score 0–4 = no extra action; 5–7 extra support in the CHC in collaboration with specialist; 8–32 assessment of specialist.

LillaLaps was found reliable, valid and suitable for recognizing children, 4- to 13-year-old, suffering from psychiatric symptoms. The internal consistency was acceptable. Sensitivity for the lower cut-off was 71% and for the higher cut-off 73%, the respective specificities being 75% and 86% [58].

The instrument was translated into Swedish by a Finnish and Swedish-speaking researcher in the project in 2019. It has not previously been used, adapted or validated for Swedish conditions. Hence, cut-off values for when support for the family should be offered are based on Finnish conditions. The questionnaire is intended to be completed by health and medical staff and is in this study used by a psychologist during assessment conversations and by the child health care nurse at the 5-year check-up.

### Template for assessment of parents experiences of neurodevelopmental problems

The template is a non-validated, clinical anamnestic guide for the CHC psychologist in the conversation with parents to preschool children referred due to neurodevelopmental problems. The template consists of 18 items to get an overall picture of the parents' experience of the child’s early development and behaviour. The CHC psychologist assessment of presence of problems are describes as 0 = not present, 1 = minor problems and 2 = severe problems. The template is in this study used to describe the neurodevelopmental problems of the included children.

### Procedures

The child health care nurse described the study to parents whose children were referred to a child psychologist for further investigation due to ESSENCE symptoms. Written information and a form for informed consent were sent to the parents before their first visit to the child health care psychologist. During the psychologist's assessment, conversation consent for contact with the preschool was obtained. The child health care psychologist obtained the clinical anamnesis according to the template and used LillaLAPS to assess the child's psychosocial health. Parents and preschool teachers assessed externalizing and internalizing behavioural problems (SDQ), including the SDQ supplement for assessing everyday function. Preschool teachers also assessed engagement and social interaction (CEQ). The children was assessed at one occasion within a period of approximately two months by the parents, the child health care psychologist and preschool teachers, respectively.

### Statistics

Statistical analyses were performed in SPSS version 25. Descriptive data are presented as mean ± SD, range, and or number/percent. Spearman rho was used to analyse correlations (mean values) between total scale LillaLAPS, CEQ, SDQ, SDQ supplement, and SDQ subscales. Student’s independent t-test was used to compare SDQ filled in by parents and preschool teachers, *p* < 0.05.

## Results

The CHC psychologist clinical anamnesis with parents showed that a majority (93%) of the parents overall were worried or reacted about the child's development, i. e ESSNCE symptoms. They experienced minor or severe difficulties to understand the child's emotional needs (54%), communication skills (71%), social ability (72%), concentration, attention and endurance (78%), activity level and impulse control (71%) and mental well-being (59%). The assessment of the CHC psychologist also revealed neuropsychiatric impairments in 55% of the families. When dividing into gender, boys showed more frequent externalizing symptoms related to ability to concentrate, attention and endurance, activity level and impulse control while girls more often presented problems with social ability, communication and difficulties with sleep, food intake and emotion regulation during infancy. Parents to boys more often expressed worries about the child’s development and well-being. They also more often declared neuropsychiatric impairments in the family, see Table 1.

**Table 1:** Assessment by child health care (CHC) psychologist's in conversations with parents of children with neurodevelopmental problems

	**Presence of neurodevelopmental problems (%)**											
	**Boys and girls**				**Boys**				**Girls**			
	n	No	Mild	Severe	n	No	Mild	Severe	n	No	Mild	Severe
Parents or others worried or reacted about the child's development	150	10 (7)	77 (51)	63 (42)	112	6 (5)	58 (52)	48 (43)	38	4 (10)	9 (30)	15 (40)
Mother had psychological or medical complications during pregnancy	147	97 (66)	38 (26)	12 (8)	111	71 (64)	29 (26)	11 (10)	36	26 (72)	9 (25)	1 (3)
Psychological or medical complications during childbirth	146	16 (80)	22 (15)	8 (5)	109	96 (79)	16 (15)	7 (6)	37	30 (81)	6 (16)	1 (3)
Difficult to understand the child emotional needs	148	68 (46)	60 (40)	20 (14)	111	52 (47)	45 (40)	14 (13)	37	16 (43)	15 (41)	6 (16)
Neuropsychiatric impairments in the family	147	66 (45)	48 (33)	33 (22)	111	49 (44)	35 (32)	27 (24)	36	17 (47)	13 (36)	6 (17)
Developmental delay during infancy	151	104 (69)	33 (22)	14 (9)	114	78 (68)	27 (24)	9 (8)	37	26 (70)	6 (16)	5 (14)
Difficulties with sleep, food intake, emotion regulation during infancy	149	86 (58)	37 (25)	26 (17)	113	70 (62)	27 (24)	16 (14)	36	16 (44)	10 (28)	10 (28)
Difficulties in gross or fine motor skills	147	110 (75)	34 (23)	3 (2)	111	80 (72)	28 (25)	3 (3)	36	30 (83)	6 (17)	0 (0)
Difficulties in communication skills	150	43 (29)	64 (42)	43 (29)	113	31 (27)	48 (43)	34 (30)	37	12 (33)	16 (43)	9 (24)
Perception deviations	151	81 (57)	52 (37)	8 (6)	108	61 (56)	41 (38)	6 (6)	33	20 (61)	11 (33)	2 (6)
Problem-solving skills in relation to age	140	121 (72)	24 (17)	15 (11)	108	80 (74)	15 (14)	13 (12)	32	21 (66)	9 (28)	2 (6)
Independence in managing daily activities in relation to age	148	85 (57)	15 (34)	13 (9)	113	64 (56)	7 (33)	12 (11)	35	21 (60)	13 (37)	1 (3)
Social ability	148	41 (28)	72 (49)	35 (23)	112	28 (25)	55 (49)	29 (26)	36	13 (36)	17 (47)	6 (17)
Ability to concentrate, attention and endurance	146	32 (22)	63 (43)	51 (35)	113	20 (18)	48 (42)	45 (40)	33	12 (36)	15 (46)	6 (18)
Activity level and impulse control	148	43 (29)	52 (35)	53 (36)	113	27 (24)	40 (35)	46 (41)	35	16 (46)	12 (34)	7 (20)
Mental well-being	147	56 (38)	56 (38)	35 (24)	111	43 (39)	44 (40)	24 (21)	36	13 (36)	12 (33)	11 (31)
Repetitive patterns in behavior, special interests, difficulty with imaginative play	148	83 (56)	54 (37)	11 (7)	112	62 (55)	41 (37)	9 (8)	36	21 (58)	13 (36)	2 (6)
Absence attacks, fluctuations in behavior and cognitive level, pronounced sleep disturbance	143	116 (82)	17 (12)	9 (6)	107	85 (79)	14 (13)	8 (8)	35	31 (88)	3 (9)	1 (3)

The CHC psychologist’s assessment with LillaLAPS confirmed ESSENCE symptoms, in terms of development related to age, everyday function, social interaction, internalizing/externalizing behavior and parents' anxiety. According to LillaLAPS, 17 children needed continued support from CHC, 32 children needed support from CHC in collaboration with specialist, and 103 children needed assessment of specialist. The mean and SD for LillaLAPS were 10 ± 4.

The combined assessment of SDQ total and SDQ supplement shows that both parents and preschool teachers estimate over the cut-off, indicating that the child has problems with behavior and everyday functions (14.0 ± 6.5 and 13.2 ± 6.2, respectively). Except for hyperactivity, there were significant differences in assessment of the different subscales. Parents assess that the child has more conduct (3.6 ± 2.4) and emotional symptoms (2.2 ± 2.3) at home, but shows more social skills (6.4 ± 2.5), compared to the preschool teachers' rating (3.0 ± 2.6, 1.6 ± 1.8 and 4.6 ± 3.1 respectively). Preschool teachers score more peer problems compared to parents' rating (3.0 ± 2.1 vs 2.5 ± 2.0). See Table 2.

**Table 2:** Cut-off, number of children above cut-off, mean ± SD (independent t-test) for SDQ (subscales, total difficulties scale, supplement) for 151 children assessed by parents and preschool teachers

	**Cut-off** **Parent/Preschool**	**n above cut-off** **Parent/Preschool**	**Parent** **m + SD**	**Preschool teacher** **m + SD**
SDQ Total difficulties scale	13/11	88/99	14.0 ± 6.5	13.2 ± 6.2
SDQ Hyperactivity^a^	6/5	75/104	5.7 ± 2.9	5.7 ± 2.9
SDQ Conduct^a^	4/3	31/76	3.6 ± 2.4	3.0 ± 2.6*
SDQ Peer	3/3	66/86	2.5 ± 2.0	3.0 ± 2.1*
SDQ Emotional	3/3	52/34	2.2 ± 2.3	1.6 ± 1.8**
SDQ Prosocial	6/4	102/90	6.4 ± 2.5	4.6 ± 3.1**
SDQ Supplement^b,c^	1/1	128/132	2.9 ± 2.4	2.4 ± 2.3

^*^p < 0.05

^**^p < 0.01

^a^Parents = 151

^b^Parents = 148

^c^Preschool teachers = 151

Mean and SD for CEQ was 2.7 ± 0.7 and median 2.7. Seventy-seven children scored above mean. The children’s engagement was scored as engaged quite often in 22.3%, and engaged very often in 32.6%.

Correlations between SDQ (total scale, subscales and supplement) and CEQ assessed by parents, preschool teachers and CHC psychologist are presented in Table 3.

**Table 3:** Correlations (Spearmans Rho) between SDQ^a^ (total sum, subscales, supplement) and CEQ^b^ assessed by parents, preschool teachers and CHC^c^ psychologist

	SDQ^a^	Preschool teacher								CHC^c^ psychologist
		SDQ^a^							CEQ^b^	LillaLAPS
		Total SDQ	Hyperactivity	Conduct problems	Peer problems	Emotional problems	Pro-social	Supplement		
Parents	Total SDQ	0.21**							0.18*	0.44**
	Hyperactivity		0.50**						0.09	0.37**
	Conduct, problems			0.17*					0.34**	0.27**
	Peer problems				0.42**				-0.29**	0.34**
	Emotional problems					0.35**			0.32**	0.19*
	Prosocial						0.46**		0.35**	-0.30**
	Supplement							0.11	0.08	0.27**
Preschool teachers	Total SDQ								-0.44**	0.33**
	Hyperactivity								-0.28**	0.23**
	Conduct, problems								-0.13	0.22**
	Peer problems								-0.66**	0.19*
	Emotional problems								-0.09	0.14
	Prosocial scale								0.68**	-0.24**
	Supplement								-0.38**	0.16
	CEQ	0.68**								-0.17*

^*^p < 0.05

^**^p < 0.01

^a^The Strengths and Difficulties Questionnaire

^b^Child Engagement Questionnaire

^c^Child Health Care

There was a positive correlation between LillaLAPS and SDQ total scored by parents and preschool teachers (0.44 and 0.33 respectively, *p* < 0.01). There was also a significant positive correlation between parents' and preschool teachers' scoring on SDQ total and SDQ subscales, but not for the SDQ supplement. LillaLAPS, screened by child psychologists, correlated positively with parental rating in the SDQ regarding hyperactivity (0.37, *p* < 0.01), conduct problems (0.27, *p* < 0.01), peer problems (0.34, *p* < 0.01), emotional symptoms (0.19, *p* < 0.05), and the SDQ supplement (0.27, *p* < 0.01), and negatively on the prosocial behaviour scale (-0.30, *p* < 0.001). LillaLAPS correlated positively with the preschool teachers' rating in the SDQ regarding hyperactivity (0.23, *p* < 0.01), conduct problems (0.22, *p* < 0.01), peer problems (0.19, *p* < 0.05), emotional symptoms (0.14, NS), and the SDQ supplement (0.16, NS), and negatively on the prosocial behaviour scale (-0.24, *p* < 0.01).

There was a negative correlation between the CEQ scored by preschool teachers LillaLAPS (-0.17, *p* < 0.05) scored by CHC psychologists respectively. The CEQ scored by preschool teachers significantly correlated negatively with SDQ total, the subscales hyperactivity and peer problems, and the SDQ supplement, but positively with the SDQ prosocial behaviour scale (0.68, *p* < 0.01). There was a positive significant correlation between CEQ and SDQ total and SDQ subscale, except for peer problems (0.29, *p* < 0.01), assessed by parents, see.

## Discussion

**Fig. 1:**
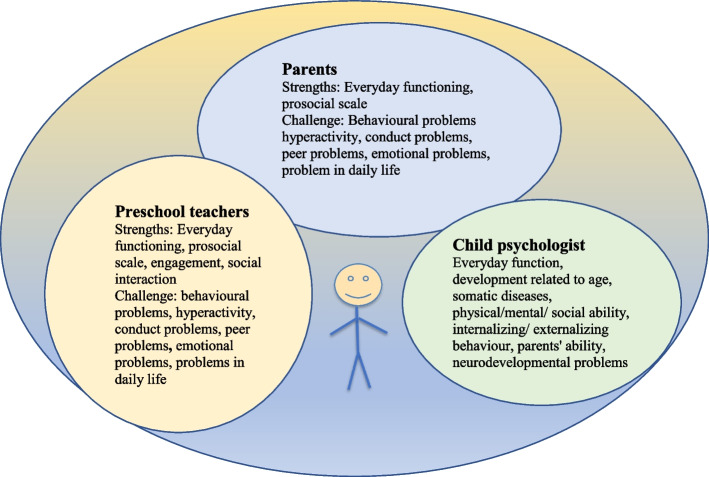
An overall picture of the child's behavior based on the assessments of the parents, preschool teachers and the child psychologist

The goal being to facilitate positive development in the child by means of early promotion, prevention and intervention [23, 59–61], which requires the cooperation of the adults who are in the child's different contexts. The present study shows that assessment of the child's behavior by parents and preschool teachers using the SDQ, teachers' assessment of engagement and social interaction using the CEQ, and the CHC psychologist's assessment of the child's psychosocial health using LillaLAPS largely agree, in terms of the child's mental health, behavioural problems and everyday function. The results also highlight the importance of considering the parents', the preschool teachers' and the child health care professional's (i.e. CHC psychologist) assessment in order to obtain an evaluation of the child's need for support in everyday life, and to detect children with ESSENCE symptoms early on. Figure 1 illustrates the overall picture of the child's behavior based on the assessments of the parents, preschool teachers and the child psychologist.

Although statistically significant, the correlations between SDQ and CEQ assessed by parents, preschool teachers and CHC psychologist in the present study are quite low. LillaLAPS correlated with the preschool's SDQ total and all SDQ subscales, except for emotional problems indicating that the CHC psychologist's anamnesis with the parents includes the child's behavior both in preschool and at home. It is reasonable to assume that, in the conversation with the CHC psychologist, the parents convey the child's situation both in the home and the preschool context.

The majority of the children scored a need for further investigation due to ESSENCE symptoms, i.e. development related to age, everyday function, social interaction, emotional behavior and parents' anxiety, assessed using LillaLAPS. The domains in LillaLAPS appear to be similar to the SDQ with the supplement, scoring behavioural problems and everyday function, and the CEQ, scoring social interaction and engagement. High scores with LillaLAPS correlated with low function in everyday life in the home environment but not in preschool. Possible explanations are the structure in preschools and that the relationship between children and adults is not as close as between children and parents, plus preschool teachers are focused more on interaction with the group than with the individual child [40].

Although the picture of the child's behavioural problems is consistent, there are differences between the parents' and the preschool teachers' assessment, which can be explained by the fact that the child is assessed in different contexts. Both parents and preschool teachers see a strength but also limitations in the child's social ability and function in daily life. Based on the SDQ cut-off s, the preschool teachers also consider hyperactivity, conduct problems and peer problems as a greater challenge compared with the parents' assessment Fälth et al. [62] showed poor coherence in SDQs filled in by parents and preschool teachers, respectively. However, in line with the present study, the subscales hyperactivity, peer problems and prosocial scale correlated highly. The varying perceptions of the child's abilities can beexplained by the fact that the child is in different contexts, in interaction with people with different demands and expectations. The family context may be affected by the parents' mental health, socioeconomic condition, other mother tongue and cultural contexts [63–65] this may also affect what the parents tell the CHC psychologist about their child's mental psychosocial health. It is important to gather information about the child in several contexts to early on detect neurodevelopmental symptoms. The challenge in clinical practice is to accept and integrate data from several sources, maybe especially the parents' concerns and description of the child's behavior.

The results indicate that both preschool teachers and parents see behavioral symptoms that noticeable affect everyday function. One explanation for the parents' appreciation of the problems in everyday life being higher compared to preschool teachers may be different roles, knowledge and experience of children's development. Preschool staff are trained in pedagogy, have more experience of children and can possibly normalize behaviours that parents perceive as problematic [62]. In addition, preschool teachers consider the child more as part of the group, and see their task as supporting the group as a whole, while the parents see the individual child. In a dissertation, Gustafsson [29] showed that when preschool teachers used the SDQ they were able to notice and understand the child’s behaviour at an earlier stage. They also found it easier to communicate concerns to the parents; something which may potentially contribute to earlier detection and help for children who need special support.

The parents' and preschool teachers' assessment of the child using SDQ total and SDQ subscales, except for the prosocial behaviour scale, are consistent with a previous review that examined psychometric properties of the SDQ in children aged 4 to 12 years [66].

The present results from the assessment with SDQ and the CHC psycologist template shows a more frequent externalizing behavior among boys compared to girls with neurodevelopmental problems. These differences are supported in other studies, reporting boys to have significantly more behavior problems than girls with the exception of the emotional SDQ subscale. Boys are also considered to have significantly lower prosocial skills than girls [29]. This may be an indication that different cut-off limits should be used for each gender. Wright et al. [67] claim that SDQ as a single report should not be relied upon as a sole means of identifying mental illness. They also argued for lower cutoff scores than advised in scoring guidance, which is supported by Silva et al. [68]. The fact that about 40% of the children in the present results scored below cutoff for SDQ total difficulties scale, might support this suggestion.

Also, one may ask how the scoring of children is affected by preschool teacher’s gender. Øvergaard et al. [69] found that the SDQ Hyperactivity subscale scored by preschool teachers was useful only in identifying girls. In addition, pre-school teachers are predominantly female (96%), which may affect expectations of behavior differently for girls and boys [29]. There is also reported that preschool teachers pay more attention to hyperactive children who disturb the group, i.e. those with externalizing problems, than on internalized behavior [29, 70].

Even if the child is hyperactive, it seems that high engagement and social interaction help them to function well [29]. However, children with low engagement and social interaction, alone or in combination with hyperactivity and conduct problems, continue to have problems, including decreasing engagement over time. Engagement improves the child's self-regulation [14], and facilitates both the child's learning [16] and well-being. In line with previous research [14, 71], we recommend to use screening instruments that assess different aspects of mental health to obtain a nuanced picture of the child. Also, it is crucial to encourage high engagement and social interaction with peers and teachers, to enhance protective factors that generate positive spirals of good mental health [14, 71].

Children with internalizing problems may worry preschool teachers, but they can more easily "handle" the child when it is quiet and inward-looking and does not disturb the group [49]. Internalizing behaviour may also be difficult to identify among preschool children, due to insufficiently developed verbal and cognitive skills. Preschool children instead use psychomotor skills to express internalization problems, such as clinging to adults [29]. Taken together, there is a risk that children who do not show behavioural problems and hyperactivity, but have other mental illness (e.g. anxiety-related symptoms) will not receive help and support in time or be missed during preschool. Teachers also often miss these symptoms in school-age children [72]. Ezpeleta et al. [60] pointed out that including ratings performed by parents enhanced detection of internalizing behaviour. In the present results, though, this did not appear as the parents did not rate emotional problems above cut-off.

In the present study, LillaLAPS did not correlate with preschool teachers' assessment of SDQ emotional symptoms. Nevertheless, children whom the CHC psychologist assessed with LillaLAPS as having behavioural problems also had low scores for engagement (e.g. in play and social interaction) in preschool. This further illuminates the importance of not only paying attention to the active, externalizing child, but also the internalizing child who withdraws from peers and, for example, prefers to play by themselves. The negative correlations between LillaLAPS and preschool teachers' rating can be interpreted as an effect of the preschool environment. Although LillaLAPS is a promising alternative for the CHC psychologist, in Sweden, it has only been used for children who are part of the PLUSS project within Jönköping County. Swedish guidelines recommend implementing valid clinical instruments like LillaLAPS that, however, could be supplemented with other assessments, such as SDQ and CEQ, as shown in the present study [73].

In the present results, the rating of engagement and social interaction according to the CEQ was lower than a normal population of children in Sweden (3.2 + 0.61) [29]. This can be explained by the fact that the children in this study have been identified with ESSENCE symptoms, which can affect social interaction and engagement. Preschool teachers' assessment with the CEQ is in line with previous research that found that children with comorbid difficulties, such as hyperactivity and conduct problems, have lower engagement, less social interaction, and more peer problems that affect everyday functioning in the preschool context [29, 57, 74]. Our conclusion is that the CEQ may be an alternative to detect children with internalizing behaviour as it focuses on engagement and interaction with peers and preschool teachers.

In order to detect children with behavioural problems earlier and counteract stigma [75], the regular health visits by the CHC nurse would be suitable occasions for screening mental health. In addition, interprofessional collaboration is of great importance in the assessment of the child, including the parents [29, 76]. Fält et al. [62] reported that preschool teachers want to identify children with problems to ensure the best interests of the child, but it emerged that both CHC nurses, parents and the preschool teachers doubted whether there was a reliable way to assess the mental health of preschool children. A barrier, may also be uneasy feelings about the parents' reactions and concerns about assessment, as well as fear of stigmatizing the child [77]. Standardized instruments for assessment, instead of subjective opinions, could remove preschool teachers' fear of stigmatizing the child [3–5, 29, 34].

## Conclusion

The present study shows that it is important to include clinical anamnesis and different validated assessment forms, filled out by different informants, to gain as complete a picture as possible of the child's health. It explores that parents, preschool teachers and child psychologists combining assessment with SDQ with impact supplement, CEQ and LillaLAPS of preschool children with ESSENCE symptoms largely agree, in terms of mental health, behavioral problems and every-day function. Also, the results highlight the importance to include the child’s different daily life context in order to identify the child's need for support in everyday life.

### Limitations

There are some limitations to consider. The study includes few girls compared to boys. However, it is a known fact that more boys than girls exhibit ESSENCE symptoms. This study could not prove girls ESSENCE symptoms specifically, nor any differences between gender. LillaLAPS is a relatively new instrument for CHC psychologists assessing psychosocial health in preschool children [50, 58], though it has to be further tested and validated in a Swedish population before implementation as a clinical assessment instrument. Also, there is a risk that assessments may be influenced by personal perceptions and preconceived expectations. It is also a limitation that we compare correlation between different instruments. This may explain relatively low significant correlations. Furthermore, it is a strength that the parents' concerns are taken seriously, but if the parents have their own mental illness, addiction, socioeconomic conditions and cultural contexts that affect their story, there may then be a risk that the child's own problems will not be detected in time.

### Clinical implications

CHC have the ability to verify neurodevelopmental problems in children at a primary level. It is important to collect information about the child in its different contexts together with the clinical anamnesis, and that CHC concern both parents and the preschool teacher’s seriously for early detection. The validated instrument SDQ and CEQ can be implemented as screening instruments in different contexts by CHC, to promote earlier detection of ESSENCE symptoms in preschool children. Screening instruments by the CHC nurse would probably lead to referring to the CHC psychologist early on. Using validated instruments in collaboration would promote detection of children not already identified as presenting neurodevelopmental problems, and hence prevent mental illness later in life.

### Future research

Lilla LAPS needs to be validated in Swedish context. Studies focusing on girls with ESSENCE symptoms and differences between boys and girls and investigating indicators of mental illness in preschool children are needed. Further studies to improve cooperation between stakeholders is needed. The inclusion of social workers in future studies can further deepen the knowledge of how parents, preschool, CHC and social care can work together when the child shows ESSENCE symptoms.

## Data Availability

All data generated or analysed during this study are included in this published article. Additional data are available from the corresponding author on reasonable request.
